# Do South Asian women with PCOS have poorer health-related quality of life than Caucasian women with PCOS? A comparative cross-sectional study

**DOI:** 10.1186/1477-7525-8-149

**Published:** 2010-12-20

**Authors:** Georgina L Jones, Manisha Palep-Singh, William L Ledger, Adam H Balen, Crispin Jenkinson, Michael J Campbell, Hany Lashen

**Affiliations:** 1 Senior Lecturer, Health Services Research Section, ScHARR, Regent Court, 30 Regent Street, Sheffield, S1 4DA, UK; 2 Consultant Gynaecologist & Subspecialist in Reproductive Medicine & Surgery, Saint Mary's University Teaching Hospital & CMMC NHS Trust, Manchester, M13 0JH, UK; 3 Professor, Academic Unit of Reproductive & Developmental Medicine, Jessop Wing Hospital, Tree Root Walk, Sheffield, S10 2TJ, UK; 4 Professor, United Leeds Teaching Hospitals, Clarendon Wing, Leeds General Infirmary, LS2 9NS, UK; 5 Professor, Department of Public Health, University of Oxford, Old Road Campus, Headington, Oxford, OX3 7LF, UK; 6 Professor, Medical Statistics Unit, ScHARR, Regent Court, 30 Regent Street, Sheffield, S1 4DA, UK; 7 Senior Lecturer, Academic Unit of Reproductive & Developmental Medicine, Jessop Wing Hospital, Tree Root Walk, Sheffield, S10 2TJ, UK

## Abstract

**Background:**

Polycystic ovary syndrome (PCOS) is the most common chronic endocrine disorder affecting women of reproductive age. This study aimed to compare the HRQoL of South Asian and white Caucasian women with PCOS, given that it is particularly common among women of South Asian origin and they have been shown to have more severe symptoms.

**Methods:**

The Polycystic Ovary Syndrome Questionnaire (PCOSQ) and the Short Form-36 (SF-36) were administered in a cross-sectional survey to 42 South Asian and 129 Caucasian women diagnosed with PCOS recruited from the gynaecology outpatient clinics of two university teaching hospitals in Sheffield and Leeds. Additional clinical data was abstracted from medical notes. Normative data, collected as part of the Oxford Health and Lifestyles II survey, was obtained to compare SF-36 results with ethnically matched women from the general UK population. Using the SF-36, normative HRQoL scores for women of South Asian origin were lower than for Caucasian women. Given this lower baseline we tested whether the same relationship holds true among those with PCOS.

**Results:**

Although HRQoL scores for women with PCOS were lower than normative data for both groups, South Asian women with PCOS did not have poorer HRQoL than their Caucasian counterparts. For both the SF-36 and PCOSQ, mean scores were broadly the same for both Asian and Caucasian women. For both groups, the worst two HRQoL domains as measured on the PCOSQ were 'infertility' and 'weight', with respective scores of 35.3 and 42.3 for Asian women with PCOS compared to 38.6 and 35.4 for Caucasian women with PCOS. The highest scoring domain for South Asian women with PCOS was 'menstrual problems' (55.3), indicating best health, and was the only statistically significant difference from Caucasian women (p = 0.01). On the SF-36, the lowest scoring domain was 'Energy & Vitality' for Caucasian women with PCOS, but this was significantly *higher *for Asian women with PCOS (p = 0.01). The best health status for both groups was 'physical functioning', although this was significantly lower for South Asian women with PCOS (p = 0.005). Interestingly, only two domains differed significantly from the normative data for the Asian women with PCOS, while seven domains were significantly different for the Caucasian women with PCOS compared to their normative counterparts.

**Conclusions:**

The HRQoL differences that exist between South Asian and Caucasian women in the general population do not appear to be replicated amongst women with PCOS. PCOS reduces HRQoL to broadly similar levels, regardless of ethnicity and differences in the normative baseline HRQoL of these groups.

## Background

Polycystic ovary syndrome (PCOS) is the most common endocrinological problem affecting women [1] with a prevalence estimated at 4-25% depending on the diagnostic criteria used [2,3]. Patients with PCOS demonstrate a combination of characteristics which may include anovulation, oligo or amenorrhoea, hirsuitism, acne, evidence of increased serum androgen levels and morphological changes in the ovary evident on ultrasonography. Diagnostically, current practice uses criteria agreed in Rotterdam 2003 [4]. Approximately 50% of PCOS patients are obese [5]; a much higher prevalence than the general population. There is also a metabolic element to the condition in the form of insulin resistance that may result in long-term morbidity.

South Asian refers to those persons who originate from the Indian subcontinent (India, Pakistan, Sri Lanka, Bangladesh and Nepal) [6]. In a community-based study in the United Kingdom (UK), it was found that polycystic ovaries (PCO) were particularly common among women of South Asian origin (52%) [7], compared to the prevalence of PCO observed in a predominately Caucasian population (22%) [8]. The South Asian population, in general, also exhibit a higher prevalence of insulin resistance and type 2 diabetes [9], which may increase long term morbidity among those with PCOS. Recent research indicated higher insulin concentrations and lower insulin sensitivity in South Asian women with PCOS compared to Caucasian women with PCOS [10]. This research also concluded that South Asians presenting with anovular PCOS were significantly younger, had more severe hirsuitism and a higher prevalence of acanthosis nigricans than their Caucasian counterparts.

Health-related quality of life (HRQoL), is a concept used to describe the physical, social and emotional effects of a disease and its associated treatments [11]. Research has shown a reduction in the HRQoL of women with PCOS compared with healthy controls [12-15]. Comparisons with other medical conditions and gynaecological populations have also yielded particularly low scores on psychological well-being and quality of life for women with PCOS [13-16].

Overall, there has been a great paucity of research comparing the influence of ethnicity or cultural background on HRQoL in women with PCOS. Two studies have tentatively explored this relationship. A study comparing Brazilian women with PCOS living in Brazil with Austrian women living in Austria found that, with the exception of body weight, the Brazilian women had worse HRQoL [17]. However, this was inter-country research and the impact of cultural, social and economic differences between the two countries is unexplained. An intra-country study of Muslim immigrants in Austria identified worse HRQoL in PCOS Muslim immigrant women compared with their Austrian counterparts [18]. However, the number of Muslim women was very small-just 14 individuals - and no attempt was made to control for any pre-existing differences in HRQoL of the groups investigated. Only one study to date has measured the HRQoL of a sample of Indian women with PCOS and found high prevalence of psychological distress in over 50% of the women as measured using the Goldberg's General Health Questionnaire-28 (GHQ-28) [19].

Twenty percent of the world's population are South Asian [6]. Figures from the 2000 census revealed that there were 1.6 million South Asians living in the United States (0.7%) [20]. In 2001, 1 million South Asians were living in Canada (3%) [21], and in 1999, a further 1 million living in Australia (1.3%) [22]. In the UK, 5.7% of the population of England and Wales identify themselves as Asian or Asian-British [23]. In 2001, 4.0% of the population were South Asian, comprising the largest minority ethnic group [24]. Because of this, it is essential to understand better the impact of PCOS in South Asian women to ensure clinical treatments are well-aligned to need.

The aim of this study was therefore to compare the HRQoL of South Asian and Caucasian women with PCOS, given that it is particularly common among women of South Asian origin and they have been shown to have more severe symptoms. Our hypothesis was that South Asian women from the Indian subcontinent with PCOS would show overall lower HRQoL than Caucasian women with the condition.

## Methods

Ethical approval for this study was obtained from the local research ethics committees of South Sheffield and Leeds West.

The sample was recruited from all women diagnosed with PCOS attending the outpatient gynaecology clinics in Sheffield and Leeds from 2003 to 2006. In Sheffield, subjects were also recruited through an existing PCOS database. Ethnicity was established from the patient's medical records and recorded against the classification recommended by the Office for National Statistics [23].

Body mass index (BMI), and evidence of polycystic ovaries (PCO) on ultrasound, testosterone concentration and acne were also recorded from the patient's medical notes. For the purposes of this study, women with PCOS were classified as having a BMI either lower than or exceeding 27.5 kg/m^2 ^to explore differences in risk profile. This is lower than the standard classification of obesity as BMI > 30 in a Caucasian population. However, this cut-off of 27.5 kg/m^2 ^is recommended to reflect the elevated risks of type 2 diabetes and cardiovascular disease for Asian populations [25,26]. Women were excluded from the study if they had another major illness that substantially influenced their quality of life or another cause of androgen excess, e.g. androgen-secreting tumours.

Patients eligible for the study were sent a covering letter, information sheet and consent form to return if they wished to participate. It has been argued that it is best to use more than one type of questionnaire when measuring health status and ideally this should comprise a generic and disease specific questionnaire. This is so comparisons can be made at a generic level and specifically to that disease [27]. Consequently, two health-related quality of life questionnaires were administered to each woman who had consented to participate; the disease-specific Polycystic Ovary Syndrome Questionnaire (PCOSQ) [28] and the generic Short Form-36 (SF-36) [29].

The PCOSQ is currently the only validated, disease-specific questionnaire available to measure the HRQoL of women with PCOS [30-32] and was administered to understand in more detail women's experiences of PCOS symptoms. The PCOSQ contains 26 items, measuring five areas of HRQoL: 'emotions' (8 items e.g. moody as a result of having PCOS?), 'body hair' (5 items e.g. growth of visible hair on chin?), 'weight' (5 items e.g. had trouble dealing with your weight?), 'infertility problems' (4 items e.g. concerned with infertility problems?) and 'menstrual problems' (4 items e.g. irregular menstrual periods?).

The SF-36 is a well validated and widely used generic health status tool [29] and was used to capture HRQoL data that could be compared to the general population. The SF-36 contains 36 items that divide into nine areas of HRQoL: 'general health perceptions' (5 items), 'change in health' (1 item), 'physical functioning' (10 items), 'role limitation due to physical factors' (4 items), 'role limitation due to emotional factors' (3 items), 'bodily pain' (2 items), 'social functioning' (2 items), 'mental health' (5 items) and 'energy and vitality' (4 items). Normative data, collected as part of the Oxford Health and Lifestyles II survey, was obtained (*personal communication with Professor Crispin Jenkinson*) to compare our patient sample to SF-36 results for ethnically matched women from the UK general population [33]. The data was selected based upon female sex and known ethnicity that was either Asian or Caucasian.

### Analysis

The age and testosterone concentration of Asian and Caucasian women were compared using independent t-tests. Chi-squared and Fisher's exact test were used to analyse data on PCO appearance on ultrasound and BMI > 27.5 kg/m^2^.

PCOS women in each ethnic group were compared to the normative SF-36 data using a two tailed t-test. The percentage difference (difference between the study sample and normative data sample means, divided by the normative data sample means) in SF-36 score relative to the general populations was calculated to highlight the magnitude of the differences for the PCOS samples.

Each item of the PCOSQ has a 7-point scale (7 represents optimal function and 1 the poorest). As with the SF-36, within each domain the sum score of the component PCOSQ questions were recoded on a scale of 0-6 and then transformed to create a total range of 0 to 100. Responses to the SF-36 domains were calculated into a range of 0-100 representing worst to best health status.

All statistical analyses were performed using SPSS version 14. The p-values are unadjusted for multiple testing.

## Results

A total of 171 women with confirmed PCOS completed the PCOSQ and SF-36 from 258 women contacted by post and an additional number recruited directly from outpatient gynaecology clinics. Of these, 42 (24.6%) were South Asian and 129 (75.4%) were Caucasian. The South Asian population consisted predominantly of women of Pakistani or Indian origin, either first or second generation, with 1% of Bangladeshi origin. The average ages of the South Asian and Caucasian women with PCOS were very similar with means of 30.0 yrs (range 20-42) and 30.3 yrs (17-48) respectively (P = 0.60).

The testosterone concentration, evidence of PCO on ultrasound and the number with a BMI > 28 kg/m^2 ^are given in Table 1. In our sample, BMI did not significantly differ between the South Asian and Caucasian women with PCOS. However, both our samples had high proportions of women exceeding the BMI cut-off of 27.5 kg/m^2 ^with 72% and 73% of the samples respectively exceeding this level. The only significant difference between the two ethnic groups was a greater likelihood of polycystic ovarian appearance confirmed on ultrasound in Caucasian women (p = 0.04).

**Table 1:** Clinical Features of the Patient Sample

	Total Patients	Asian	Caucasian	Two-tailed significance
Sample size	171	42	129	

BMI >27.5 kg/m^2^	106 (72.6%)	28 (71.8%)	78 (72.9%)	p = 0.90

PCO on Ultrasound	143 (93.5%)	29 (85.3%)	114 (95.8%)	p = 0.04*

Testosterone ng/dl: Mean (SD)	2.37 (0.90)	2.59 (0.70)	2.30 (0.95)	p = 0.12

* (Fisher's Exact Test)

### The SF-36

On the SF-36, the lowest mean total score and thus worst area of HRQoL for the South Asian group was observed for 'general health' (53.9) (Table 2). Within the Caucasian group the mean total score was lowest for 'energy and vitality' (45.4). The highest and therefore best health status for both groups was 'physical functioning', although this was significantly lower for South Asian women with PCOS (73.0) than for Caucasian PCOS women (87.3) (p = 0.005). 'Energy and vitality' was the only other domain to show a significant difference between the two groups (p = 0.01). Other domains were more similar and without a consistent direction of difference.

**Table 2:** Comparison of the Asian PCOS and Caucasian PCOS mean SF-36 domain scores.1

SF-36	Asian PCOS Mean	Caucasian PCOS Mean	Difference	95% CI	Two tailed significance
Bodily Pain	61.4 (n = 42)	67.3 (n = 126)	5.9	-3.5 to 15.3	p = 0.22

Energy and Vitality	54.9 (n = 40)	45.4 (n = 127)	9.5	-16.9 to -2.0	p = 0.01

General Health	53.9 (n = 40)	57.8 (n = 125)	4.0	-4.0 to 11.9	p = 0.33

Mental Health	60.0 (n = 41)	57.6 (n = 127)	2.4	-9.6 to 4.8	p = 0.52

Physical Functioning	73.0 (n = 39)	87.3 (n = 124)	14.4	4.5 to 24.1	p = 0.005

Role Limit/Emotion	64.3 (n = 42)	53.9 (n = 125)	10.4	-24.6 to 3.8	p = 0.15

Role Limit/Physical	67.3 (n = 42)	77.6 (n = 126)	10.3	-1.9 to 22.5	p = 0.10

Social Functioning	64.9 (n = 41)	65.3 (n = 127)	0.3	-9.7 to 10.3	p = 0.95

^1^. 0 (Indicating Worst Health Status) to 100 (Best Health Status)

### The PCOSQ

With the PCOS-specific instrument, the South Asian women's lowest mean PCOSQ scores and thus worst area of HRQoL were in the domains of 'infertility' (35.3) and 'weight' (42.3) (Table 3). These were also the lowest scoring domains for Caucasian women (38.6 and 35.4 respectively). The highest scoring domain for South Asian PCOS was 'menstrual problems' (55.3), indicating best health, and was the only statistically significant difference from Caucasian women (p = 0.01). In contrast, 'body hair' (54.9) was the highest scoring domain for Caucasian women with PCOS.

**Table 3:** Comparison of the Asian PCOS and Caucasian PCOS mean PCOSQ domain scores.1

PCOSQ	Asian PCOS Mean	Caucasian PCOS Mean	Difference	95% CI	Two tailed significance
Body Hair	50.8 (n = 41)	54.9 (n = 126)	4.1	-7.8 to 16.0	p = 0.50

Emotions	51.0 (n = 36)	51.9 (n = 120)	0.8	-8.7 to 10.4	p = 0.86

Infertility	35.3 (n = 41)	38.6 (n = 121)	3.3	-7.7 to 14.3	p = 0.55

Menstrual Problems	55.3 (n = 41)	43.5 (n = 121)	-11.8	-20.6 to -3.0	p = 0.01

Weight	42.3 (n = 40)	35.4 (n = 125)	-6.9	-18.6 to 4.9	p = 0.25

^1^. 0 (Indicating Worst Health Status) to 100 (Best Health Status)

### Comparison with the general population

Data from the Oxford Health and Lifestyles survey were used to indicate normal non-PCOS SF-36 scores for the population within each ethnic group. This survey includes 57 Asian women and 4897 Caucasian women. This is the only normative data readily available, and does have weakness in that it includes older age women. Whereas the mean age of each PCOS group was 30 years, the survey data means were 35.8 yrs for Asian women (p < 0.01) and 39.6 yrs (p < 0.01) for Caucasian women. The limited sample size for the Asian population responses means that matching methodologies, on age or other characteristics, were not possible

Asian women in the general population reported significantly lower and therefore worse HRQoL on all SF-36 domains compared with their Caucasian counterparts (Table 4). On every domain, HRQoL scores were lower for South Asian and Caucasian women with PCOS compared with equivalent normative data for their ethnicity (i.e. negative percentage difference in Table 4). However, South Asian women with PCOS showed only small decreases in HRQoL from the normative data in several domains; e.g. 'energy and vitality' and 'physical functioning' were 0.02% and 7.2% lower respectively. The only two statistically significant HRQoL differences for South Asian PCOS women were for 'social functioning' (p = 0.01) and 'general health' perceptions' (p = 0.02), which were 17.8% and 16.4% lower than the normative data. None of the domain means for the South Asian women with PCOS were more than 20% lower than the Asian general population.

**Table 4:** Percentage difference in HRQoL for PCOS women relative to the SF-36 scores from the normative data

SF-36 Domain	* Normative Asian mean	South Asian PCOS (% difference from normative Asian)	P value	* Normative Caucasian mean	Caucasian PCOS (% difference from normative Caucasian)	P value
Bodily Pain	69.8 (n = 57)	-12.0	p = 0.12	79.6 (n = 4834)	-15.5	p < 0.01

Energy and Vitality	55.0 (n = 51)	-0.02	p = 0.98	58.9 (n = 4769)	-23.0	p < 0.01

General Health	64.5 (n = 57)	-16.4	p = 0.02	73.8 (n = 4751)	-21.7	p < 0.01

Mental Health	67.7 (n = 56)	-11.4	p = 0.08	72.3 (n = 4737)	-20.0	p < 0.01

Physical Functioning	78.7(n = 53)	-7.2	p = 0.31	87.8 (n = 4650)	-0.06	p = 0.06

Role Limit/Emotion	72.7 (n = 55)	-11.6	p = 0.28	81.0 (n = 4801)	-33.5	p < 0.01

Role Limit/Physical	79.8 (n = 52)	-15.7	p = 0.09	84.2 (n = 4801)	-7.8	p = 0.01

Social Functioning	79.0 (n = 54)	-17.8	p = 0.01	86.8 (n = 4836)	-24.8	p < 0.01

NB. SF-36 range: 0 (Worst Health Status) to 100 (Best Health Status)

* Data obtained from the Oxford Health and Lifestyles II survey from a personal communication with Professor Crispin Jenkinson.

In contrast, seven statistically significant HRQoL differences for Caucasian women with PCOS were observed and they showed lower HRQoL of 20% or more from the normative data in five domains, with the biggest differences being 'role limitations-emotional' and 'social functioning' showing 33.5% (p < 0.01) and 24.8% (p < 0.01) lower HRQoL.

## Discussion

The aim of this paper was to compare the HRQoL of South Asian and Caucasian women diagnosed with PCOS. Existing research has identified a younger age of presentation with oligomenorrhoea, increased hirsutism, acne, acanthosis nigricans and infertility in South Asian women from the Indian subcontinent with PCOS as compared with Caucasian women with PCOS [7,10,34]. For this reason, we anticipated that the HRQoL scores of South Asian women with PCOS would be worse than for Caucasian women with the condition. This expectation was reinforced by knowledge from the Oxford Health and Lifestyles Survey II that showed lower HRQoL among Asian women generally.

Overall, rather than finding consistent differences, we found the HRQoL of Asian and Caucasian women with PCOS to be broadly similar when measured with the condition-specific questionnaire (PCOSQ). The only domain of the PCOSQ to show a significant difference between the two groups was 'menstrual problems', which was found to be of least concern for Asian women with the condition. Wijeyratne et al [10] found that Asian women began experiencing oligomenorrhoea at a younger age. This significant difference may be the result of a longer period of adjustment to the menstrual problems for the Asians and/or earlier presentation to specialist services. However, a further longitudinal study would be needed to explore this hypothesis.

Consistent with other cross-sectional studies of women with PCOS [12,13,30,31,35,36], we found that weight and infertility were the worst domains on the PCOSQ for both ethnic groups. This is perhaps not surprising given that the metabolic profile of the condition means that is very difficult for a PCOS woman to lose weight. Indeed, with the exception of surgery, interventions to lose weight are often unsuccessful and associated with high rates of weight regain [37]. Thus it is perhaps not surprising that many PCOS women typically report frustration in losing weight, low self-esteem and consequently a poor body image [28]. In relation to weight, a poor body image in PCOS women may be compounded by cultural influences as it has been shown that android fat pattern, commonly associated with PCOS, is considered unattractive in many cultures [38,39]. However, other explanations particularly found in Western cultures regarding women, for example societal expectations of thinness, may also be responsible.

Higher mean scores (indicating a better HRQoL) were found on the SF-36 compared with the PCOSQ, probably because of the generic nature of the questionnaire: one limitation of using generic measures is that they may not be sensitive enough to assess changes in specific illnesses as they are designed to measure HRQoL across a wide variety of diseases [27]. Questionnaires designed specifically for patients with a given disease should be more responsive or sensitive to changes in health status because they contain items from relevant patient groups. Asian women with PCOS scored worse than Caucasian women with PCOS on the 'physical functioning' and 'role limitation: physical' domains. However, these had relatively high HRQoL scores for both groups, which is consistent with other research showing it is the physical domains of the SF-36 where PCOS has the least impact [13]. In contrast, 'Energy and Vitality' was the lowest scoring SF-36 domain for Caucasian women with PCOS women and also significantly lower than for Asian women with the condition.

The results from both PCOSQ and SF-36 lead us to conclude that HRQoL is not significantly worse for Asian women with PCOS than it is for Caucasian women with PCOS. In fact, the only significant differences between the two groups in domains where PCOS symptoms have a major impact, actually showed worse HRQoL for Caucasian women ('menstrual problems' on the PCOSQ and 'energy and vitality' on the SF-36). This result is surprising, since the SF-36 normative data showed Asian women had significantly poorer HRQoL scores than Caucasian women. Tentatively, it suggests that serious health disorders might reduce HRQoL to similar levels, regardless of starting HRQoL and overrides less severe HRQoL factors.

There are a number of limitations to this research. Our study sample data were obtained from women with PCOS attending outpatient gynaecology clinics and therefore are limited to those PCOS women who attend a clinic, rather than a wider population including women with PCOS not having treatment. The normative data with which comparison is made were collected in 1993 and did not contain any finer level ethnicity data than 'Asian'. This broad ethnic grouping will mean that it may not be a precise match for those South Asian women with PCOS from the Indian sub-continent that we recruited in clinic, especially as they were also significantly older.

While this is the largest study to date that has compared the HRQoL of PCOS women from different ethnicities, it needs to be recognised that a sample of 42 Asian PCOS women and the small normative data set for the Asian population is not optimal. Recruitment of minority ethnic women proved problematic in our study as reported in other forms of health research [40], and highlights how any advances in engaging such groups to participate would be beneficial.

A high level of obesity is found in both Caucasian and South Asian populations. The traditional cut-off points for BMI are overweight (25 kg/m^2 ^< 30 kg/m^2^) and obese as (> 30 kg/m^2^). However, these related to risk thresholds for mortality and morbidity in mainly European populations [41]. Increasingly, evidence suggests that even with a low BMI (< 25 kg/m^2^), South Asian's suffer an increased risk of hypertension, diabetes and dislipidemia and therefore the BMI thresholds have been lowered to 23 kg/m^2 ^(overweight) and 25 kg/m^2 ^(obese) to reflect this risk for this population [25]. Recent publications have also stressed the need to measure beyond BMI and also measure waist circumference; it is more directly proportional to total body fat and the amount of metabolically active visceral fat and therefore is a more accurate measure of metabolic risk [42]. Recently, the waist circumference threshold has also been reduced to reflect a high risk profile to >/= 80 cm for Asian Indian women [26]. Thus in future studies of ethnicity, PCOS and HRQoL, the relationship between this parameter and HRQoL outcomes may give a more accurate picture of the role of weight upon a woman's health status, especially given the confusing and contentious state of using different BMI cut-offs for different ethnicities.

## Conclusion

PCOS has a major negative impact upon HRQoL, regardless of whether women are from an Asian or Caucasian ethnic background, with weight and infertility worst affected by the condition. But although there is evidence of poorer HRQoL for Asian women in the general population than for Caucasian women, Asian women do not seem to have identifiably poorer HRQoL with PCOS than Caucasian women with PCOS. This suggests that effective management of HRQoL of Asian women with PCOS may not differ from more mainstream practices.

## Competing interests

The authors declare that they have no competing interests.

## Authors' contributions

GJ conceived of the study, participated in its design, performed the statistical analysis and drafted the manuscript for publication. MS conceived of the study, participated in its design and assisted with recruitment. WL conceived of the study and participated in its design. AB conceived of the study and participated in its design. CJ provided and assisted with the analysis of the SF-36 normative data. MJC assisted with the statistical analysis. HL participated in the study design and assisted with recruitment. All authors read and approved the final manuscript.
